# Neural Dynamics of Inhibitory Control in Musicians with Absolute Pitch: Theta Synchrony as an Oscillatory Signature of Information Conflict

**DOI:** 10.1093/texcom/tgab043

**Published:** 2021-07-03

**Authors:** Vivek V Sharma, Michael Thaut, Frank A Russo, Claude Alain

**Affiliations:** Neurosciences and Mental Health Program, Hospital for Sick Children, Toronto, ON M5G 0A4, Canada; Music and Health Sciences, Faculty of Music, University of Toronto, Toronto, ON M5S 2C5, Canada; Department of Psychology, Ryerson University, Toronto, ON M5B 2K3, Canada; Music and Health Sciences, Faculty of Music, University of Toronto, Toronto, ON M5S 2C5, Canada; Rotman Research Institute, Baycrest Health Sciences, Toronto, ON M6A 2E1, Canada; Department of Psychology, University of Toronto, Toronto, ON M5S 3G3, Canada; Institute of Medical Sciences, University of Toronto, Toronto, ON M5S 1A8, Canada

**Keywords:** stroop, oscillations, music, EEG, cognition

## Abstract

Absolute pitch (AP) is the ability to identify an auditory pitch without prior context. Current theories posit AP involves automatic retrieval of referents. We tested interference in well-matched AP musicians, non-AP musicians, and nonmusicians with three auditory Stroop tasks. Stimuli were one of two sung pitches with congruent or incongruent verbal cues. The tasks used different lexicons: binary concrete adjectives (i.e., words: *Low*/*High*), syllables with no obvious semantic properties (i.e., solmization: *Do*/*So*), and abstract semiotic labels (i.e., orthographic: *C*/*G*). Participants were instructed to respond to pitch regardless of verbal information during electroencephalographic recording. Incongruent stimuli of words and solmization tasks increased errors and slowed response times (RTs), which was reversed in nonmusicians for the orthographic task. AP musicians made virtually no errors, but their RTs slowed for incongruent stimuli. Frontal theta (4–7 Hz) event-related synchrony was significantly enhanced during incongruence between 350 and 550 ms poststimulus onset in AP, regardless of lexicon or behavior. This effect was found in non-AP musicians and nonmusicians for word task, while orthographic task showed a reverse theta congruency effect. Findings suggest theta synchrony indexes conflict detection in AP. High beta (21–29 Hz) desynchrony indexes response conflict detection in non-AP musicians. Alpha (8–12 Hz) synchrony may reflect top-down attention.

## Introduction

Musicians with absolute pitch (AP) can name the pitch-class of a musical tone in less than a second without the aid of an external referent. Most musicians cannot do this (Baharloo et al. 1998) and require an external pitch and referent in short-term memory to name a subsequent pitch before the memory trace vanishes. This latter ability, called relative pitch (RP), is based on knowledge of pitch-class intervals acquired during music training. While RP can be learned at any age, AP acquisition is observed early in life and thought to involve an automatic mapping of acoustic pitch to morphemes, similar to that observed for object naming during language acquisition (Takeuchi and Hulse 1993; Russo et al. 2003; Vitouch 2003; Miyazaki and Ogawa 2006). Indeed, there is evidence that AP automaticity seems to be mediated by strong verbal associations to pitch, which may be difficult to suppress (Akiva-Kabiri and Henik, 2012). Notably, musicians with AP and RP show increased grey matter density in language and auditory areas, implying a possible link between verbalization and music training (Gaser and Schlaug 2003; Dohn et al. 2015). While current models of AP posit strong automatic association between pitch and verbal label, the nature of this association in AP is still debated.

Verbal labels can be made to interfere with response goals in the auditory Stroop task, where participants are asked to respond to the pitch of a congruent stimulus (e.g., the word “low” in low voice) or an incongruent stimulus (e.g., the word “low” in higher voice). Incongruent stimuli tend to produce increased response times (RTs) and reduced response accuracy, which is referred to as Stroop interference effect. Past studies have identified two neural correlates of Stroop task interference effects: The N450 is a negative going modulation that increases at 450 ± 100 ms after the onset of an incongruent stimulus and has been proposed as an index of conflict detection. This neural correlate is followed by a late positive component in the 600–900 ms poststimulus interval thought to index conflict resolution (West and Alain 1999).

Prior studies using these neural correlates as a metric of Stroop interference effect have shown comparable amplitude modulation in musicians with or without AP (Itoh et al. 2005; Greber and Jäncke 2020). Sharma et al. (2019) used three different versions of the Stroop tasks: A word version in which concrete congruent and incongruent adjectives were presented (e.g., the word low in low pitch voice vs. low in high pitch voice), a solmization version in syllables with no obvious semantic properties were presented (i.e., *Do*/*So*), and an orthographic version in which abstract semiotic labels were presented (i.e., *C*/*G*). They showed that N450 indexed Stroop interference effects in AP in the word and orthographic version of the Stroop tasks, whereas non-musicians (NMs) and musicians with RP showed a clear N450 modulation only in the typical word version of the Stroop task. These results support a small advantage of AP in noticing conflict compared to NMs or musicians with RP. However, it is possible that analyses limited to the time-domain only, such as in event-related potentials analyses, may have missed important information. Electroencephalogram (EEG) analyses that take into account spectral power differences and nonphase locked activity (i.e., time-frequency analyses) may be better suited to identify neural correlates of conflict detection and conflict resolution in musicians with or without AP.

In particular, theta (4–7 Hz) power peaking between 400 and 500 ms after stimulus onset over frontal scalp areas has been associated with Stroop interference effects (Botvinick et al. 1999; Szűcs and Soltèsz 2012; McDermott et al. 2017). Studies have also reported enhanced delta (1–3 Hz) power for congruent stimuli over parietal region between 300 and 600 ms after stimulus onset (Ergen et al. 2014). Interestingly, there is evidence that AP have increased left hemispheric theta phase coupling between prefrontal and auditory areas (Elmer et al. 2015). Further, one study reported increase theta power during stimulus and response conflict in the Stroop task, while high beta (20–30 Hz) power was found for response conflict independent of stimulus conflict (Zhao et al. 2015).

In the present study, we used time–frequency analysis to investigate neural dynamics of interference in AP. Well-matched NMs, RP-musicians, and AP-musicians were analyzed for main effects or interactions between group and congruency effect in three variations of the Stroop task described earlier (Sharma et al. 2019). We predicted that theta power would significantly increase over frontal scalp areas during information conflict, especially during the word Stroop. We also hypothesized that musicians with AP will show theta congruency effects regardless of lexicon, while non-AP musicians would show congruency effects only during the words version of the Stroop task. Furthermore, we anticipated increased suppression of high beta power for incongruence across groups, reflecting conflict between stimuli and response mappings. Finally, we hypothesized that the magnitude of inhibitory control would be proportional to semantic information in the stimuli.

## Method

### Participants

Musicians were defined as individuals with a minimum of 7 years formal lessons and NMs as individuals with a maximum 3 years of formal lessons. They were recruited from the Faculty of Music at University of Toronto, and completed a music aptitude test aiming to identify those with AP or RP. Musicians who reported having AP were first given the AP screening test (see below). If they passed the test, then they were included in the AP group, otherwise they were assigned to the RP group and asked to complete the RP screening test (see below). Musicians who did not report having AP were given the RP test first. Rapid and highly accurate scores on the RP test, and also reported early age piano training, were also screened for AP. If these participants passed the AP screening test, then they were assigned to the AP group. Two musicians originally tested with the RP test, also passed the AP test.

Twenty NMs (aged between 20 and 39, 5 males), 20 musicians with AP (aged between 19 and 41, 5 males), and 20 musicians with RP (aged between 21 and 39, 5 males) were originally recruited for behavioral and EEG analysis. AP and RP did not significantly differ in the amount of musical training years, years of education, self-reported handedness, and age. One NM, one AP musician, and one RP musician were excluded from the analysis due to excessive artifacts resulting in poor signal-to-noise ratio.

Ethics approval and informed consent were obtained from all participants according to the guidelines set out by the Research Ethics Board of Baycrest Hospital and the University of Toronto.

### Screening for Music Aptitude

The screen tests employed the same computer and digital audio system as the experiment. The participants were instructed to click the center circle to hear a tone for AP or two consecutive tones for RP. The RP musicians were further instructed that they would always hear C4 before a second pitch, which they should then label. The participants were then instructed to click within the circle that contains the correct pitch-class label using the mouse as rapidly and accurately as possible. Each of these regions displayed a complete chromatic pitch-class label, including enharmonic equivalents. Participants were scored for the percentage of correctly labeled tones. For the purposes of this study, the criterion for AP will be strictly defined as the ability to score ≥80% or a mean absolute deviation of ≤1 under these screening test conditions (Bermudez and Zatorre 2009). RP was strictly defined as the ability to score ≥80% and a mean absolute deviation score of ≤1. RTs that were 3 standard deviations from the group mean were eliminated. A set of structured questionnaires and follow-up interviews were then administered after testing to gather and index musical experience, language, and demographic data.

### Experimental Tasks

#### Stimuli

The stimuli analyzed in this study were sung tones comprising of monosyllabic morphemes that were either semantically congruent or incongruent to pitch: /low/,/high/,/do/,/so/,/see/, and, /jee/. A professional female vocalist sung the phonemes, which were recorded at a sample rate of 44 100 Hz using a Shure SM58 microphone and ProTools LE (Version 7.3.1, Daly City, CA).

The fundamental frequency of each tone was transformed digitally to be tuned to 261.63 Hz for the low pitch and 392 Hz for the high pitch using Adobe Audition 1.5 (2004). Pitch tuning of analog signal was verified with a Korg CA-20 tuner using European Harmonized Standard at A4 = 440 Hz. The stimuli were edited to have a 500 ms duration including 5 ms rise/fall time. All phonemes were standardized to the Carnegie Melon University Pronouncing Dictionary. Stimuli were presented binaurally at an intensity of 75 decibels sound pressure level through insert earphones model ER-3A by Etymotic Research (Elk Grove Village, 1985). Intensity calibration was confirmed with a model AEC201 ear simulator and sound level meter system by Larson Davis (Depew, 2008).

#### Procedure

Participants sat in an acoustically and electrically shielded room in a cushioned armchair. The Stroop task paradigm was programmed using Presentation 16.5 software (Neurobehavioral Systems Inc.). Each trial began with the presentation of a white fixation cross. After 500 ms, an auditory stimulus was presented (500 ms in duration). The white fixation cross was maintained on the computer screen black background throughout the stimulus presentation, and postresponse for 500 ms, after which the next trial proceeded. The intertrial interval was 1000 ms. Each block of trials comprised 100 congruent and 100 incongruent of a single lexicon, as well as 67 neutral stimuli. Three blocks of trials were completed by the participants and breaks were offered between the blocks. Participants were instructed to press the “Left Arrow” for “Low” pitch and the “Right Arrow” for “High” pitch, regardless of sung word. RTs were defined as the time between the onset of an auditory stimulus and the instance of a key press. Participants were asked to respond as rapidly and accurately as possible. Responses that were less than 200 ms or greater than 1000 ms poststimulus presentation were removed from the analysis as false-alarms or slow responses. Error rates are the proportion of incorrect responses for each participant.

#### E‌EG Recording

EEG was recorded from 66 scalp electrodes using a BioSemi Active Two acquisition system (BioSemi V.O.F., Amsterdam, The Netherlands). The electrode montage was based on the 10/10 system and included a common mode sense active electrode and driven right leg passive electrode serving as ground. Ten additional electrodes were placed below the hair line (both mastoid, both preauricular points, outer canthus of each eye, inferior orbit of each eye, two facial electrodes) to monitor eye movements and to cover the whole scalp evenly. Neuro-electric activity was digitized continuously at rate of 512 Hz with a bandpass of DC-100 Hz, and stored for offline analysis. All off-line analyses were performed using Brain Electrical Source Analysis software (BESA, version 6.1; MEGIS GmbH, Gräfelfing, Germany).

The continuous EEG data were first digitally filtered with 0.5 Hz high-pass (forward, 6 dB/octave) and 80 Hz low-pass filters (zero phase, 24 dB/octave). A multiple source eye correction algorithm was used for ocular artifact removal, which has been shown to produce effective corneoretinal correction (Berg and Sherg 1994; Dimigen et al. 2011; Kornrumpf et al. 2016). For each participant, a set of brain topographies were identified in the continuous EEG recording as surrogate models of spatial components that best account for eye movements. The spatial topographies were then subtracted from the continuous EEG to correct for lateral and vertical eye movements as well as for eye-blinks. The analysis epoch consisted of 1000 ms of prestimulus activity and 1000 ms of poststimulus activity time-locked to the stimulus onset. After correcting for eye movements, all experimental files for each participant were then scanned for artifacts using a peak-to-peak window; epochs that contained deflections exceeding ±60 μV were marked and excluded from the analysis. The remaining epochs were averaged across electrode position, stimulus type (i.e., congruent, incongruent), lexicon (i.e., Words, Solmization, Orthographic). Trials containing responses less than 200 ms or greater than 1000 ms poststimulus were removed from analysis. Only correct response trials were included in the analysis.

**Figure 1:**
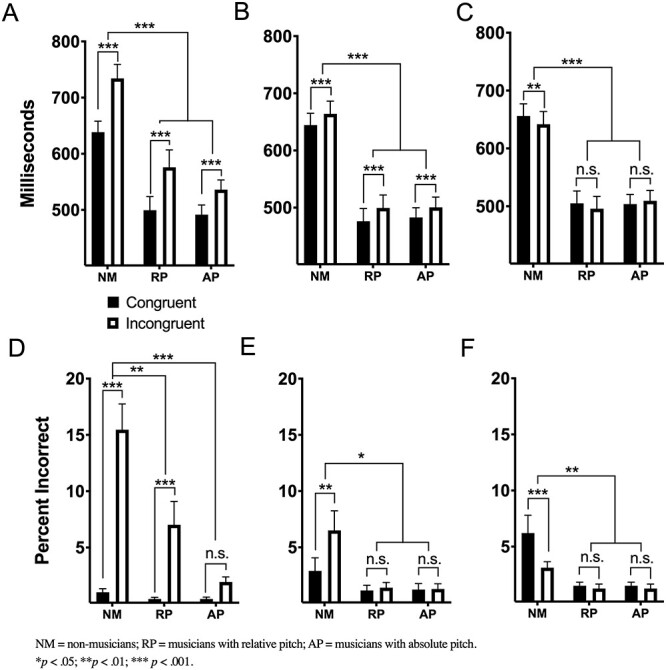
Behavioral results of nonmusicians (NM), AP musicians and RP musicians in three tasks with different lexicons. Musicians are more rapid and accurate, with greater resistance to information conflict. In (*A*), musician RTs are significantly faster than NM for the words task, but all groups are susceptible to congruency effect. The same is true of the solmization task in (*B*), yet the interference is smaller in magnitude. For the orthographic task in (*C*), NM show a reverse congruency effect but neither musician group is significantly slower when actively suppressing incongruent orthographs. Error rates in (*D*) show AP is highly precise and not susceptible to error in the Stroop task. This is true for both musician groups for musical labels as seen in (*E*) and (*F*).

#### Time–Frequency and Statistical Analysis

Time–frequency analysis was completed on Brain Electrical Source Analysis (BESA) software (version 7). A complex demodulation method was used for decomposing the single trial EEG data into time-frequency representation with 1 Hz wide frequency bins ranging from 2 to 60 Hz, and 50 ms wide time bins. The calculation method resulted in what are referred to as event-related spectral perturbations (ERSPs) through quantification of the temporal spectral evolution (TSE) of power changes relative to a baseline interval ranging from –200 to 0 ms prestimulus presentation. Positive differences (enhancement) are referred to as event-related synchronization (ERS), while negative differences (suppression) are referred to as event-related desynchronization (ERD). We defined the frequency bands of interest as delta (2–3 Hz), theta (4–7 Hz), alpha (8–12 Hz), low beta (13–20 Hz), high beta (21–29 Hz), beta rebound (15–29 Hz), and low gamma (30–60 Hz). Statistical analyses were done separately in two time windows: 350–550 ms and 600–900 ms. Power from these time–frequency windows were averaged across a frontocentral region of interest consisting of FC1, FCz, FC2, C1, Cz, and C2 scalp electrodes. This amounted to two time–frequency-electrode windows for each band that were held constant across factors that were statistically analyzed with a full factorial mixed design repeated-measures ANOVA in SPSS. Behavioral results from Sharma et al. (2019) were refactorized exclusively with current independent variables. The within-subjects factors were: Lexicon (i.e., words, solmization, and orthographic) and stimulus type (i.e., congruent or incongruent). The between-subjects factor was group (i.e., NM, RP, and AP). Post hoc tests employed Bonferroni procedure and Geisser-Greenhouse correction was applied where applicable.

## Behavioral Results

Figure 1 shows the group mean RTs and error rates as a function of the Stroop tasks. The three-way interaction between group, lexicon, and stimulus type was significant for RT (*F*(4, 108) = 7.30, *P* < 0.001, η_p_^2^ = 0.213) and error rate data (*F*(4, 108) = 10.24, *P* < 0.001, η_p_^2^ = 0.275). This three-way interaction was driven primarily by a reverse Stroop interference effect in the Orthographic task for NM and musicians with RP, whereas the musicians with AP tended to be slower for incongruent than congruent stimuli. AP error rates were unaffected by Stroop interference during incongruent words and solmization. A comprehensive behavioral analysis can be found in Supplemental Material 1.

## Neurophysiological Results

**Figure 2:**
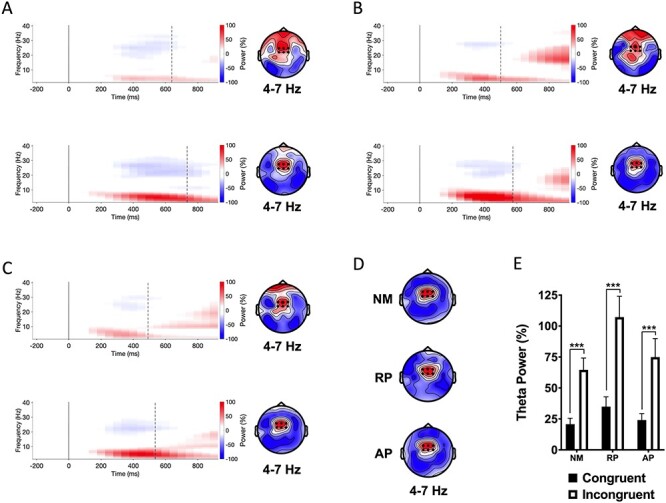
Event-related spectral perturbations (left) and topographic maps (right) of theta power between 350 and 550 ms poststimulus onset for auditory Stroop task. The solid vertical line in spectrograms marks the center of the time bin at stimulus onset and the dashed black line represents group mean RTs. (*A*) Nonmusician temporal spectral evolution for congruent (top panel) and incongruent stimuli (bottom panel) averaged across six electrodes displayed as dots on the topographic maps. The topographic maps show mean power for 350–550 ms interval. (*B*) Musicians with RP. (*C*) Musicians with AP. (*D*) Topographic maps showing differences of theta event-related synchrony for each group. (*E*) Group mean power for the 350–550 ms interval averaged across six electrodes displayed as black dots on the contour maps. NM = nonmusicians; RP = musicians with relative pitch; AP = musicians with AP. ^*^^*^^*^P < 0.001.

**Figure 3:**
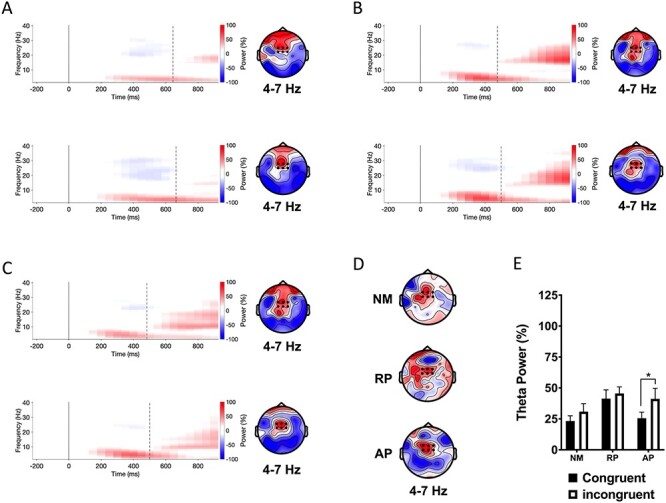
Event-related spectral perturbations (left) and topographic maps (right) of theta power in response to lexical items from solmization (i.e., solfège) version of the Stroop task. (*A*) Nonmusician spectrograms averaged across six electrodes (black dots) displayed on the topographic maps for congruent (top panel) and incongruent stimuli (bottom panel). Topographic maps show group mean theta power for the 350–550 ms interval. (*B*) Musicians with RP. (*C*) Musicians with AP. (*D*) Topographic maps showing differences of theta event-related synchrony for the 350–550 ms interval. (*E*) Group mean theta power for the 350–550 ms interval averaged across six electrodes (black dot) for congruent and incongruent stimuli. NM = nonmusicians; RP = musicians with relative pitch; AP = musicians with AP. ^*^P < 0.05.

**Figure 4:**
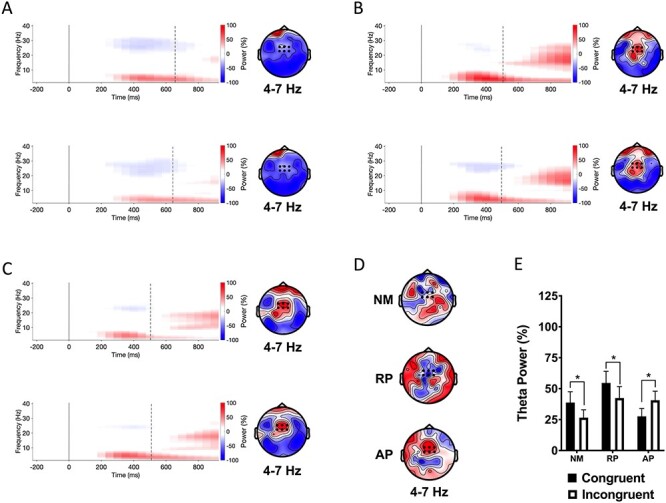
Event-related spectral perturbations (left) and topographic maps (right) from the orthographic task. Dashed black lines in the spectral plot show the RTs. (*A*) Nonmusician spectrograms for congruent (top panel) and incongruent stimuli (bottom panel) averaged across six frontocentral electrodes (black dots on the topographic maps). (*B*) Musicians with RP. (*C*) Musicians with AP. (*D*) Topographic maps showing differences of theta event-related synchrony for the 350–550 ms interval. (*E*) Group mean theta power for the 350–550 ms interval averaged across six electrodes (black dot) for congruent and incongruent stimuli. NM = nonmusicians; RP = musicians with relative pitch; AP = musicians with AP. ^*^P < 0.05.

Figures 2, 3, and  4 show the group mean ERSPs for the words, solmization, and orthographic versions of the auditory Stroop task, respectively. For the word version of the Stroop task, we anticipated greater theta power ERS for incongruent than congruent stimuli in all three groups. We also hypothesized that this neural correlate of the Stroop interference effect would be smaller for the solmization and orthographic lexicon. Lastly, we predicted that musicians with AP would show strong Stroop interference effects in all three lexicons. We also analyzed oscillatory activity within the delta and gamma bands, which can be found in Supplementary Materials 2 and 3, respectively.

### Theta Synchrony (350–550 ms)

The three-way interaction between group, lexicon and stimulus type was significant (*F*(4, 108) = 2.47, *P* = 0.049, η_p_^2^ = 0.084). The two-way interaction between lexicon and stimulus type was also significant (*F*(2, 108) = 44.33, *P* < 0.001, η_p_^2^ = 0.451). Planned comparisons show that incongruent stimuli in the word task elicited greater theta ERS power than incongruent stimuli in the solmization (*P* < 0.001) and orthographic task (*P* < 0.001). This is consistent with the general idea that using increasingly more abstract description of sound pitch yield weaker Stroop interference effects. However, congruent stimuli in the orthographic task elicited significantly greater theta ERS than congruent stimuli in the word task for NMs (*P* = 0.013) and musicians with RP (*P* = 0.010). Theta power for congruent stimuli was not significantly different across lexicon in musicians with AP (*P* = 1). To better understand this three-way interaction, the subsequent analyses examine the Stroop interference effect within each task (i.e., lexicon) separately using ANOVA.

#### Word Stroop Task

The group × stimulus type interaction was not significant, indicating comparable Stroop interference effects across groups. The main effect of group was not significant (*F*(2, 54) = 2.67, *P* = 0.078, η_p_^2^ = 0.090). There was, however, a main effect of stimulus type (*F*(1, 54) = 61.29, *P* < 0.001, η_p_^2^ = 0.532), with incongruent stimuli generating greater theta power than congruent stimuli in each group (see Fig. 2).

#### Solmization Stroop Task

The group × stimulus type interaction was not significant. There was a main effect of stimulus type (*F*(1, 54) = 6.34, *P* = 0.015, η_p_^2^ = 0.105). However, post hoc tests revealed that this was driven by AP, who showed significantly greater theta power for incongruent than congruent stimuli (*P* = 0.016). The congruency effect was not significant in NMs or RP (see Fig. 3).

#### Orthographic Stroop Task

There was a significant group × stimulus type interaction (*F*(2, 54) = 5.86, *P* = 0.005, η_p_^2^ = 0.178). NMs and musicians with RP showed reverse congruency effects such that theta power was significantly greater for congruent than incongruent stimuli (NMs: *P* = 0.048; RP: *P* = 0.047). Musicians with AP showed greater theta power for incongruent than congruent stimuli (*P* = 0.035). These results are displayed in Figure 4.

### Theta Synchrony (600–900 ms)

The effect of musicianship on conflict resolution was examined for the 600–900 ms interval. The three-way interaction was not significant. There was a significant interaction between lexicon and stimulus type (*F*(2, 108)  = 15.34, *P* < .001, η_p_^2^ = 0.221). Post hoc tests show that incongruent stimuli in the word version of the Stroop task generated significantly greater ERS across groups than incongruent stimuli in the solmization (*P* < 0.001) and orthographic tasks (*P* < 0.001).

### Alpha Synchrony (350–550 ms)

We examined whether congruency effects would also be reflected in alpha power, and whether these effects would differ as a function of group and lexicon. These analyses allowed us to examine the extent to which conflict detection is specifically indexed by changes in theta power.

**Figure 5:**
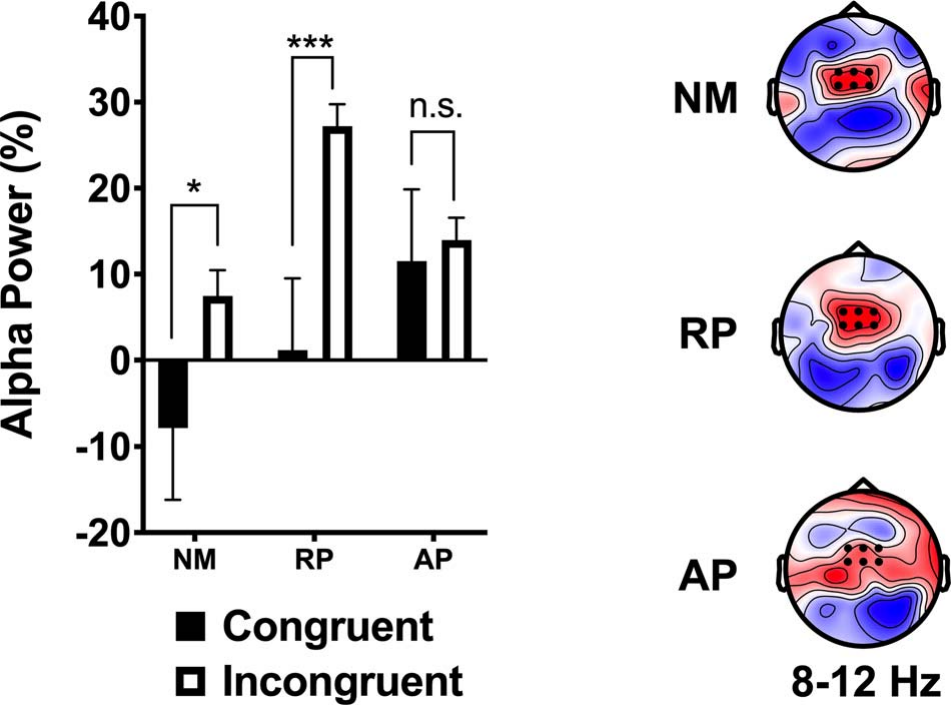
Left: Group mean alpha power (event-related synchronization) for the 350–550 ms interval in the word version of the Stroop task across six frontocentral electrodes (black dot on the topographic maps). Right: Topographic maps showing the difference in alpha power between congruent and incongruent stimuli for the 350–550 ms interval. NM = nonmusicians; RP = musicians with relative pitch; AP = musicians with AP. ^*^P < 0.05; ^*^^*^^*^P < 0.001.

The three-way interaction was not significant. A significant two-way interaction between group and stimulus type was significant (*F*(2, 54) = 3.26 *P* = 0.046, η_p_^2^ = 0.108). The two-way interaction between lexicon and stimulus type was also significant (*F*(2, 108) = 4.76, *P* = 0.010, η_p_^2^ = 0.081). There was also a main effect of stimulus type (*F*(1, 54) = 16.77, *P* < 0.001, η_p_^2^ = 0.237). Post hoc tests revealed that alpha ERS power for incongruent words was significantly greater than for congruent stimuli in NM (*P* = 0.037) and RP (*P* < 0.001) groups but not in musicians with AP (*P* = 0.735). NMs showed alpha ERD for congruent words, which was the only conditions and group to do so in this time window. RP musicians showed marginal congruency effects during the solmization task (*P* = 0.053). No significant group differences were found for congruence effects during the orthographic version of the Stroop task. Results are displayed in Figure 5.

### Alpha Synchrony (600–900)

The three-way interaction was significant (*F*(4, 108) = 2.50, *P* = 0.047, η_p_^2^ = 0.085). There was also a significant interaction between lexicon and stimulus type (*F*(2, 108) = 5.76, *P* = 0.004, η_p_^2^ = 0.119). Significantly greater alpha ERS was found for words compared to solmization (*P* < 0.001) and orthographic (*P* = 0.038) lexicons, while the latter two musical lexicons did not differ from each other. To better understand the three-way interaction, we examined the group difference in Stroop interference effects in each task separately.

#### Words Stroop Task

The interaction between group and stimulus type was significant (*F*(2, 54) = 3.45, *P* = 0.039, η_p_^2^ = 0.113). Post hoc tests showed significantly different in alpha power in NMs between congruent and incongruent stimuli (*P* < 0.001). Congruent condition elicited alpha ERS in NMs, while their incongruent alpha response showed ERD. The AP group did not show significant difference in alpha ERS power between incongruent and congruent stimuli (*P* = 0.257), nor did the RP group (*P* = 0.144).

#### Solmization Stroop Task

The main effect of group was not significant nor was the main effect of stimulus type or the interaction between group and stimulus type.

#### Orthographic Stroop Task

The main effect of group was not significant nor was the main effect of stimulus type or the interaction between group and stimulus type.

### High Beta Desynchrony (350–550 ms)

**Figure 6:**
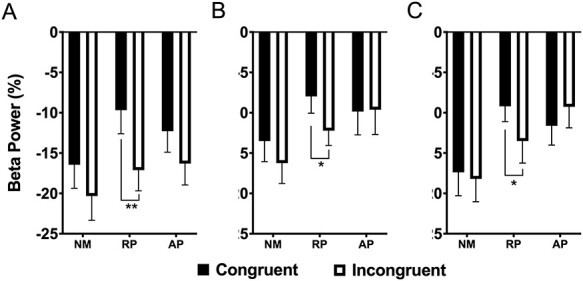
Group mean high beta power for the 600–900 ms interval across six frontocentral electrodes in the words in (*A*), solmization in (*B*) and orthographic (*C*) tasks. NM = nonmusicians; RP = musicians with relative pitch; AP = musicians with AP. ^*^P < 0.05; ^*^^*^P < 0.01.

The three-way interaction was not significant. A significant interaction was found between group and stimulus type (*F*(2, 54) = 4.40, *P* = 0.017, η_p_^2^ = 0.139), with musicians with RP showing greater congruency effect than NM or musicians with AP (see Fig. 6). There were also significant main effects of lexicon (*F*(2, 108) = 7.67, *P* < 0.001, η_p_^2^ = 0.124) and stimulus type (*F*(1, 54) = 16.86, *P* < 0.001, η_p_^2^ = 0.238). There were no significant high beta band differences between 600 and 900 ms poststimulus.

### Beta Rebound (600–900 ms)

**Figure 7:**
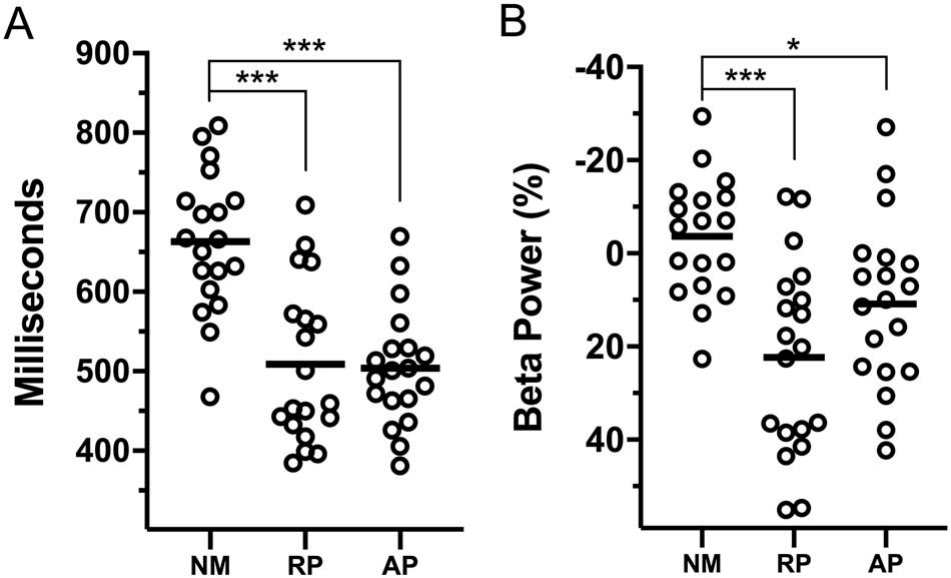
Scatterplots showing individual averages of RTs and post-response beta rebound between 600 and 900 ms poststimulus onset. (*A*) Individual mean RTs. (*B*) Beta power. NM = nonmusicians; RP = musicians with relative pitch; AP = musicians with AP. ^*^P < 0.05; ^*^^*^^*^P < 0.001.

For beta rebound power, the three-way interaction was not significant. The interaction between lexicon and stimulus type was significant (*F*(2, 108) = 12.46, *P* < 0.001, η_p_^2^ = 0.187). There was also a main effect of group (*F*(2, 54) = 10.32, *P* < 0.001, η_p_^2^ = 0.277). Post hoc tests show that NMs had significantly lower beta ERS than RP (*P* < 0.001) or AP (*P* = 0.046). Figure 7 displays beta rebound differences for each group.

**Figure 8:**
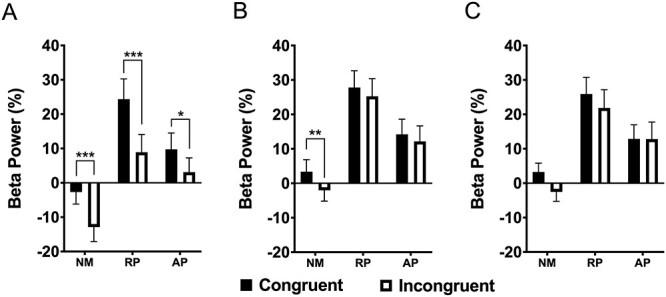
Group mean beta rebound for the 600–900 ms interval across six frontocentral electrodes. (*A*) Word task. (*B*) Solmization task. (*C*) Orthographic task. NM = nonmusicians; RP = musicians with relative pitch; AP = musicians with AP. ^*^P < 0.05; ^*^^*^P < 0.01; ^*^^*^^*^P < 0.001.

We observed greater beta ERS for congruent than incongruent words in AP (*P* = 0.025), and RP (*P* < 0.001). In NMs, ERD was lower for incongruent words than congruent words (*P* < 0.001). For solmization, NMs showed significant power differences between congruent and incongruent stimuli (*P* < 0.01). The effect of stimulus type was not significant during the orthographic Stroop task (Fig. 8).

### Correlations

**Figure 9:**
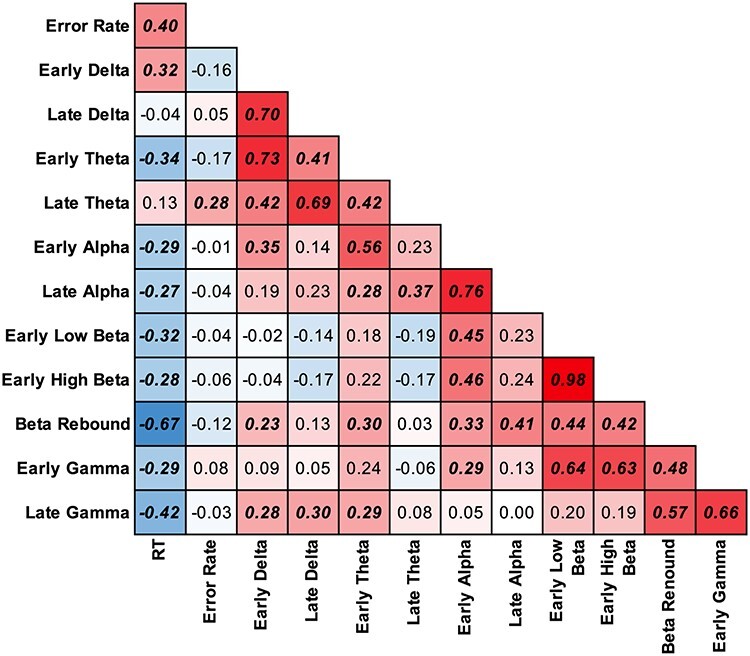
Heatmap and matrix of linear correlations for each oscillatory band per time window (Early is 350–550 ms; Late is 600–900 ms). Significant correlations are bolded and italicized.

Pearson’s correlations assessed any linear relationships between behavior and ERSPs. We expected late and early band power to correlate within bands. Notably, theta and beta bands did not correlate possibly demonstrating independent neural processes (Fig. 9). However, theta and alpha bands are positively correlated along with alpha and beta. While gamma was not analyzed by ANOVA, correlation suggests that decreasing gamma is related to increasing beta band.

## Discussion

This study aimed to better characterize the neural underpinnings of AP during Stroop interference using three tasks with differing lexicons. AP and RP musicians were significantly faster and more accurate than NMs, providing converging evidence for benefits of music training on cognitive function. All three tasks showed behavioral effects during information conflict. Stroop interference occurred for incongruent words and solmization. During the orthographic version of the Stroop task, a reverse congruency effect was found in NMs (i.e., congruent led to slower RTs), possibly due to the influence of acoustic brightness (as indexed by spectral centroid) on pitch perception (Russo et al. 2019). In particular, as shown in Table 1, C (/see/) has a higher spectral centroid than G (/jee/). AP showed resistance to Stroop interference effects, which were indexed by two brain oscillation signatures thought to reflect conflict detection (N450; 350–550 ms poststimulus) and conflict resolution (LPC; 600–900 ms, poststimulus), respectively. However, AP and RP RTs showed that their conflict resolution occurred during the N450, unlike NMs who responded during the LPC time-course. ERSPs of delta, theta, and alpha bands had relatively frontocentral scalp topographies for band-limited oscillatory power. Task-related effects are discussed below.

### Theta Power Indexes Information Conflict Detection in Absolute Pitch

In musicians with AP, the processing of incongruent stimuli was associated with enhanced theta ERS in all three versions of the Stroop task. This congruency effect on theta power also occurred in AP when they did not exhibit behavioral evidence of Stroop interference effects. Indeed, while theta power congruency effects were always consistent with information conflict in AP, it did not translate into interference at the response stage. Thus, while theta ERS seems to provide neural evidence of conflict detection in AP, this group was nevertheless able to overcome the conflict without effecting accuracy and sometimes even RT. Further, NM and RP groups showed a strong theta congruency effect for words and a reverse theta congruency effect in the orthographic version of the Stroop task. This reverse effect was consistent with NM task performance, and provides neuroelectric evidence that AP likely perceives information conflict in situations where non-AP do not. It may be that theta ERS is a generic index for information conflict of multiple types. This would also include semantic conflict from words, which may explain the profoundly enhanced theta power compared to music lexicons. Indeed, the increased inhibitory control requirements for words evidenced in behavior seem to also be indexed by theta band. Thus, theta ERS seems to index varying demand levels of Stroop tasks. This includes conflict detection in AP regardless of response (e.g., motor actions). We note that theta may also index response level conflict, however even when response conflict was not found, theta power was enhanced by congruency.

**Table 1:** Table shows verbal and spectral content. Semantic content is congruent to both pitch and spectral centroids of the words and solmization lexicons. Incidentally, semantic content is incongruent to spectral centroids of the orthographic morphemes, possibly implying the presence of a phonological incongruence in these stimuli

Lexicon	Stimulus type	Morpheme	Phonemes	Pitch-class	Pitch (Hz)	Centroid (Hz)
Words	Congruent	Low	/low/	C4	261.85	501.22
Words	Incongruent	High	/high/	C4	261.14	801.21
Words	Congruent	Low	/low/	G4	392.01	701.78
Words	Incongruent	High	/high/	G4	392.94	1187.78
Solmization	Congruent	doh	/do/	C4	261.33	425.69
Solmization	Incongruent	soh	/so/	C4	261.75	879.28
Solmization	Congruent	doh	/do/	G4	391.26	696.64
Solmization	Incongruent	soh	/so/	G4	392.32	1084.04
Orthographic	Congruent	C	/see/	C4	262.31	819.41
Orthographic	Incongruent	G	/jee/	C4	261.27	710.59
Orthographic	Congruent	C	/see/	G4	391.38	990.5
Orthographic	Incongruent	G	/jee/	G4	391.68	834.79

Interestingly, non-AP musicians also showed a reverse theta congruency effect for theta power during the orthographic task. These effects were analogous to the behavioral findings observed in NMs. This reverse congruence effect suggests an association between pitch and phoneme, albeit the opposite of what we expected. Upon examination of stimuli, we found that spectral centroids, which reflect phonemic articulation content, were greater when unvoiced phonemes were present in the stimuli (i.e., /s/ at onset of/see/). Voiced consonant onsets (i.e., /j/ at onset of /jee/) produced lower spectral centroids than unvoiced onsets. It seems that when unvoiced consonants were paired with the low pitch and a voiced consonant with the high pitch, both pairings contained incongruities. Therefore, centroids of unvoiced phoneme were congruent with pitch and label in the word and solmization tasks, but not the orthographic task. This means for congruent words and solmization stimuli, two auditory features were congruent to pitch but for congruent orthographic stimuli, only one feature was congruent to pitch (i.e., label) while the other was incongruent (i.e., unvoiced phoneme). Based on these results, it appears that the sibilant /s/ phoneme appears to associate to high pitch, and this interfered with the low orthographic congruent stimuli (i.e., /see/). Further, it seem that voiced consonants (e.g., /j/) may associate to low pitch, which interfered with high orthographic congruent stimuli (i.e., /jee/). Interestingly, theta ERS results imply that this incongruence may have been detected by RP as well. Non-AP groups may have implicitly associated two sensory percepts (i.e., pitch-class and unvoiced phoneme) more strongly than the abstract semantic level mappings learned from musical notation.

Theta ERS was sensitive to sub-lexical information conflict in non-AP. This highlights the role of implicit and explicit knowledge when determining conflict between two acoustic features, or between pitch and abstract encodings. We suggest that AP detects explicit conflict between pitch-class and verbal label, which can be evidence as increased theta power remaining across lexicons with and without inhibitory control. Further research is needed to better understand how different reference systems (i.e., articulation, spectral information, semantic association, etc.) influence task performance and neural oscillations. This peculiar reverse congruency effect in non-AP has not been described before, though the same verbal codes have been used in previous AP Stroop tasks (Itoh et al. 2005). These results are consistent with accounts that pitch-class to alphanumeric orthograph association is automatic only in AP (Zatorre et al. 1998).

Behavioral interference and theta congruence effects were both largest for auditory words. It is possible that the semantic encoding of words generates both stimulus conflict (associative perception) and response conflict (associative behavior), which sums as overall theta power. Nevertheless, we show that theta power increases in AP during information conflict without impacting speed and accuracy. Thus, theta power increases for stimulus conflict both in the presence and absence of response conflict. We propose that theta conflict effects can serve as a neural signature of perceptual conflict detection even without a concrete semantic property. This metric appears to be more effective than broadband time domain measures, which do not seem as sensitive to pitch-label conflict (Itoh et al. 2005).

Past studies suggest that theta oscillations emanate from neural assemblies in the anterior cingulate cortex (ACC), which may act as a cortical hub that facilitates information flow required for conflict detection and subsequent inhibitory control (Hanslmyer et al. 2008; Schulze et al. 2012). It may be possible that theta ERS at ACC can index multiple types of information conflict perception, possibly as a result of linking multiple networks that contribute to the Stroop task.

### Alpha Synchrony May Reflect Top-Down Auditory Attention

Alpha ERS was found across groups, tasks and time windows. However, alpha ERS was significantly decreased in musicians with RP for congruent compared to incongruent words. In NMs, alpha ERD was found for congruent words, which was significantly different than the alpha ERS found for their incongruent words. Further, NMs showed alpha ERD followed by alpha ERS for congruent words and the reverse for incongruent words. This replicates color word Stroop results attributing alpha ERD to activation facilitating semantic attention (Klimesch 1999; Ergen et al. 2014).

Previous research suggests that alpha/beta ERS reflects active neuronal inhibition, whereas alpha/beta ERD may reflect a release from this inhibition, or possibly neuronal excitation (Jensen et al. 2005; Klimesch et al. 2007; Jensen and Mazaheri 2010; Sharma et al. 2021). If this is the case, AP may maintain inhibitory processes for congruent words, while NM and RP tend to release this inhibition. In NMs, this neuronal inhibition tends to be fully relinquished at response stages for incongruent words, which would necessitate increased inhibitory requirements during response. In contrast, AP showed consistent alpha ERS in both time windows across tasks and stimuli. These results suggest that AP possessors recruit auditory attention and listening effort similarly across lexicons (Dimitrijevic et al. 2019). It may be possible that in NMs, words automatically release these inhibitory mechanisms. However, RP results imply that this may be overcome to some extent by prolonged musical training. We propose that alpha ERD indexes a prepotent and feedforward excitatory response for words that facilitates efficient semantic perception. Further, we suggest that NMs experience greater interference than RP and AP musicians for words due to increased difficulty suppressing semantic automaticity associated with contextually well-defined words related to pitch such as low and high. Suppression of this response may be indexed by alpha ERS, which seems to be a reliable index of top-down auditory attention. In the case of AP, we suspect that selective attention may be guided to internal representations of labels and alpha ERS reflects AP inhibition of semantic attention across tasks.

### Beta Desynchrony Indexes Response Conflict in Musicians with Relative Pitch

Beta ERD emerged at preresponse stages and was offset postresponse. It was prolonged during information conflict from words in NMs, which corresponded with their significantly increased RTs. While low beta only showed congruency effects for NMs with no group differences overall, a congruency effect was found for high beta ERD power only in RP. Along with the high speed and accuracy of RP group, beta ERD findings seems to be consistent Stroop task accounts concluding that beta ERD reflects response conflict independent of stimulus conflict detection (Zhao et al. 2015). We suggest that beta ERD effects are also possibly perceptual in musicians with RP, since they occurred in the absence of response inhibition.

### Beta Rebound May Reflect Motor Planning in Musicians

Enhanced postresponse beta ERS (i.e., the beta rebound) was found in musicians from 600 to 900 ms poststimulus, which occurred after keypress in musicians. NMs had typically longer RTs and sometimes showed beta ERD in this time window. This beta ERD in NMs may reflect ongoing response execution, delaying onset of the postresponse processing. Beta rebound power also paralleled the expertise related behavioral advantages found across tasks at the group level. This implies that postresponse beta synchrony is found at earlier times as a result of decreased RTs in musicians. Beta ERS has been described as a neural correlate of cortical idling and reflects neuronal inhibition in motor cortex (Pfurtscheller et al. 1996). It has also been suggested that beta rebound reflects active maintenance of a motor plan (Cao and Hu 2016; Hao et al. 2020). Perhaps, musicians speed and accuracy for this task can be attributed to more rapid maintenance of response mapping and active control over disengagement of motor system.

## Conclusions

Musicians with AP show conflict detection, as indexed by theta power, regardless of lexicon. This finding provides support for models of AP that posit strong automatic association between pitch and verbal label. Time-frequency analyses reveal more robust group differences in conflict deflection and conflict resolution than analyses limited to time-domain only. High beta ERD (suppression) increased similarly for information conflict only in musicians with RP, suggesting different neural processes than AP when resolving response conflict. However, postresponse “beta rebound” was enhanced earlier in both AP and RP musicians, which was consistent with the behavioral advantages of the musicians. Beta band may index advantages to motor systems from prolonged training of perceptuomotor skills. Thus, protracted neuroplastic changes brought about by prolonged musical training also contribute to neurophysiological and behavioral measures of cognitive inhibition in musicians with AP.

## Supplementary Material

Supplementary_Figure_1_color_tgab043

supplementary_material_review_tgab043

supplementary_material_tgab043
