# Supplementary figures and images for: Neural Dynamics of Inhibitory Control in Musicians with Absolute Pitch: Theta Synchrony as an Oscillatory Signature of Information Conflict
Source: Cereb Cortex Commun. 2021 Jul 3;2(3):tgab043. doi: 10.1093/texcom/tgab043 (PMC8423588; doi:10.1093/texcom/tgab043)

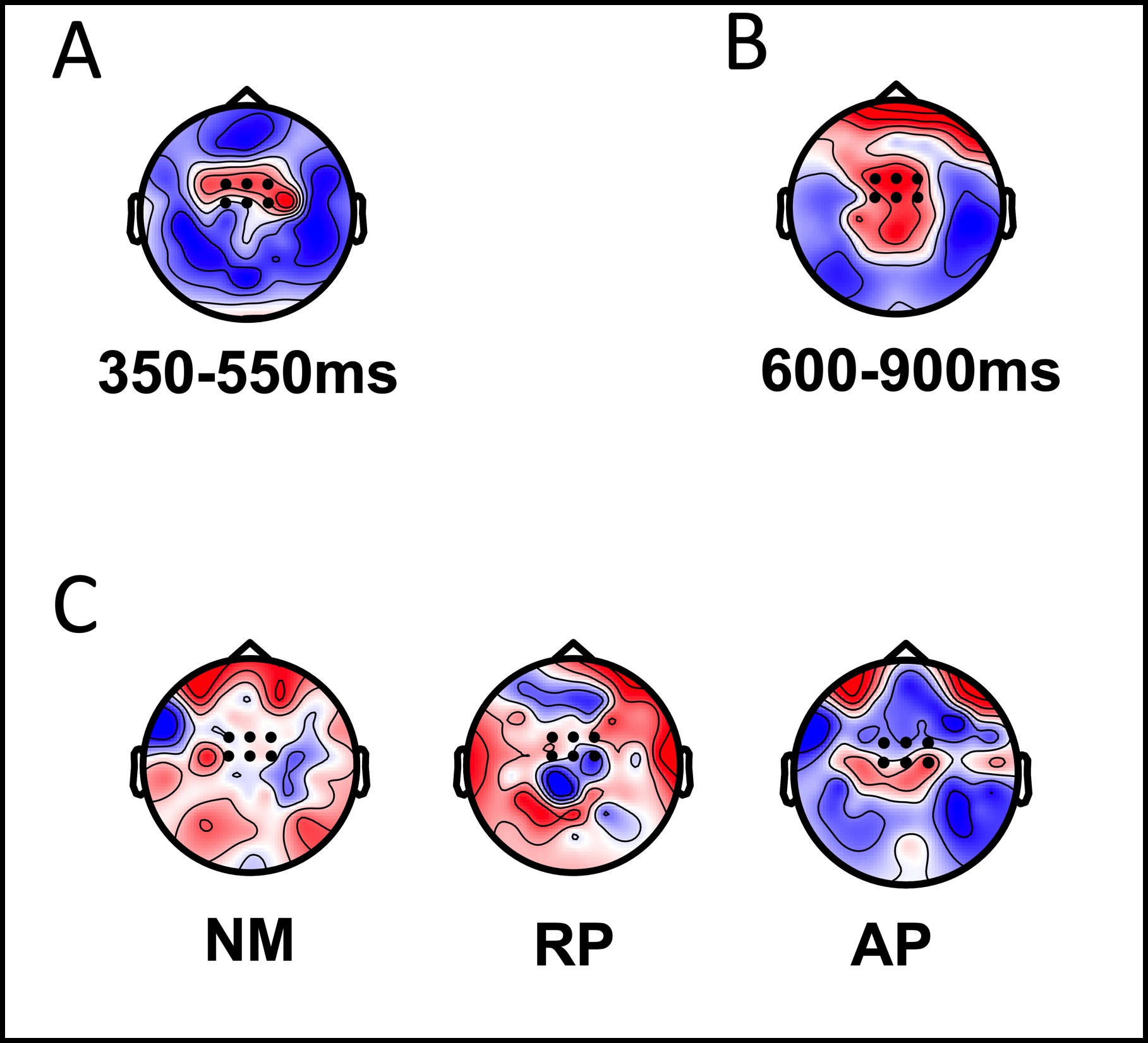

Supplement: Supplementary_Figure_1_color_tgab043 [file supplementary_figure_1_color_tgab043.jpeg]
