# Supplementary material for: Neural Dynamics of Inhibitory Control in Musicians with Absolute Pitch: Theta Synchrony as an Oscillatory Signature of Information Conflict
Source: Cereb Cortex Commun. 2021 Jul 3;2(3):tgab043. doi: 10.1093/texcom/tgab043 (PMC8423588; doi:10.1093/texcom/tgab043)
Supplement: supplementary_material_review_tgab043 [file supplementary_material_review_tgab043.docx]

Neural dynamics of inhibitory control in musicians with absolute pitch: Theta synchrony as a neural signature of information conflict (Supplementary Material)

Vivek V. Sharma^1^, Michael Thaut^2^, Frank A. Russo^3^ & Claude Alain^2,4,5,6^

^1^Neurosciences and Mental Health, Hospital for Sick Children, Toronto, ON, Canada

^2^Music and Health Sciences, Faculty of Music, University of Toronto, ON

^3^Department of Psychology, Ryerson University, Toronto, ON, Canada

^4^Rotman Research Institute, Baycrest Health Sciences, Toronto, ON, Canada

^5^Department of Psychology, University of Toronto, ON

^6^Institute of Medical Sciences, University of Toronto, ON

*Correspondence should be addressed to:

Dr. Claude Alain

Baycrest Health Sciences

3560 Bathurst Street

Toronto, ON, M6A 2E1

[calain@research.baycrest.org](mailto:calain@research.baycrest.org)

***1.1 Three-factor behavioral analysis of response times***

In the words lexicon there was a significant group × stimulus type interaction (*F*(4, 108) = 7.19, *p* < .001, η_p_^2^ = .210), along with a main effect of group (*F*(2, 54) = 17.67, *p* < .001, η_p_^2^ = .396). Post hoc comparisons show that there was no difference between AP and RP (*p* = 1) and that both musician groups were significantly faster than NMs (*p* < .001 for both differences). NMs were significantly slower for incongruent trials than congruent trials (*p* < .001), while musicians with AP and RP were significantly slower for incongruent than congruent (*p* < .001) with smaller differences between stimulus types than NMs. For the solemnization lexicon, the main effect of group was significant (*F*(2, 54) = 21.52, *p* < .001, η_p_^2^ = .444). AP and RP musicians were not significantly different from each other in RT (*p* = 1) and both musician groups were faster than NMs (*p* < .001 for both differences). There was also a main effect of stimulus type (*F*(2, 54) = 66.71, *p* < .001, η_p_^2^ = .553). Post hoc comparisons show that the congruent condition was significantly faster than incongruent (*p* < .001). For the orthographic lexicon, the interaction between stimulus type and group was significant (*F*(2, 54) = 4.65, *p* = .014, η_p_^2^ = .147). The main effect of group was again significant (*F*(2, 54) = 17.70, *p* = .001, η_p_^2^ = .396) where no difference was found between AP and RP (*p* = 1) but both musician groups were faster than NMs (*p* < .001). However, the NM groups responded significantly faster for incongruent than congruent keycodes trials (*p* < .01).

***1.2 Three-factor behavioral analysis of error rates***

For error rates in English words, the interaction between group × condition was significant (*F*(2, 54) = 12.92, *p* < .001, η_p_^2^ =.324). The NM groups were significantly less accurate than both musician groups overall in the words lexicon (*p* < .001 compared to AP and *p* < .01 compared to RP), while the musician groups were not significantly different in error rates overall for English words. Musicians with AP did not show significant differences in accuracy for congruent and incongruent English word stimuli, while the NM and RP group did show significant information conflict effects (*p* < .001 for both NM and RP). For the solemnization lexicon, a significant interaction between group × condition was found (*F*(2, 54) = 7.98, *p* < .001, η_p_^2^ = .228). The AP and RP groups were significantly more accurate than NMs in this lexicon (*p* = .022 vs. AP and *p* = .024 vs. RP) but not significantly different from each other (*p* = 1). Musicians with RP and AP showed similar error rates in this lexicon between congruent and incongruent with no significant differences, while NMs were significantly faster for congruent than incongruent solfège trials (*p* < .001). For orthographs, there was a significant group × condition interaction (*F*(2, 54) = 3.23, *p* = .047, η_p_^2^ = .107). There was also a significant main effect of group (*F*(2, 54) = 7.50, *p* < .01, η_p_^2^ = .217). AP and RP musicians were significantly more accurate overall than NMs (*p* < .01 vs. both AP and RP) but musician groups were not significantly more accurate than each other (*p* = 1). Musicians with RP and AP showed comparable error rates between congruent and incongruent stimuli with no significant accuracy differences, while NM were significantly more accurate for the incongruent than congruent conditions (*p* < .001).

***2.1 Delta (350-550ms)***

The interaction between lexicon and congruence was significant (*F*(2,108) = 6.23, *p* < .01, η_p_^2^ = .103). Post hoc tests revealed a significant increase of ERS from incongruence across groups in the word lexicon (*p* < .001). See supplemental Figure 1a.

***2.2 Delta (600-900ms)***

The interaction between lexicon and congruence was significant (*F*(2,108) = 5.33, *p* < .01, η_p_^2^ = .09). Congruent and incongruent conditions elicited significantly greater delta power for the word lexicon across groups (*p* < .001; see supplemental Figure 1b). However, delta ERS in AP also significantly increased for incongruent keycodes (*p* = .035; see supplemental Figure 1c).


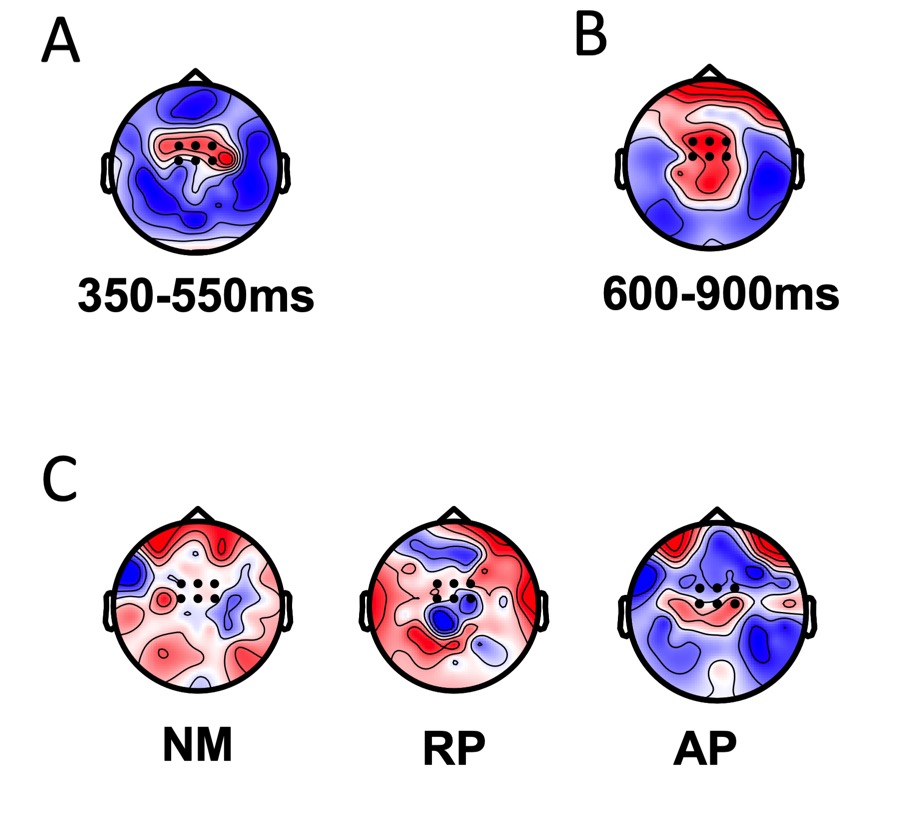


**Supplementary Figure 1.** (A) Topographical map shows delta (2-3 Hz) event-related synchrony difference for incongruent and congruent words. Black dots in map represent electrodes used for statistical testing. (B) Words elicit a significant delta band congruency effect. (C) Difference maps show only AP group has a significant congruency effect for *C* and *G* labels 600-900ms post-stimulus onset with a similar but more posterior topography as words.

***2.3 Delta Power Discussion***

The words task produced significantly greater delta ERS power during Stroop trials than the music lexicons in both time windows. This implies a prolonged delta ERS specific to a fluent language. We suggest that these differences may be related to semantic information from words. Notably, this delta congruency effect was also found in AP for the labels *C* and *G*, which interpolation shows is a slightly more posterior response.

***3 Low Beta Desynchrony (350-550ms)***

The three-way interaction was not significant. There is a significant interaction between group and stimulus type (*F*(2, 108) = 14.03, *p* < .001, η_p_^2^ = .206). Beta ERD power was greater in congruent than incongruent stimuli in musicians with RP during the word (*p* < .01) and orthographic tasks (*p* = .022). The word task showed greater low beta ERD than the solmization (*p* < .01) and orthographic tasks (*p* = 0.041). There was also a main effect of lexicon (*F*(2, 108) = 8.59, *p* < .001, η_p_^2^ = .137).

***4.1 Gamma (350-550ms)***

No significant results.

***4.2 Gamma (600-900ms)***

There was a main effect of group (*F*(2, 54) = 4.22, *p* = .02). Post hoc tests show that the RP group showed significantly greater gamma ERS than NMs (*p* = .022). AP showed an intermediate value between NM and RP groups that did not significantly differ from either group.

***4.3 Gamma Power Discussion***

Gamma ERS was increased in RP and AP compared to NM. A possible low gamma enhancement might be associated to musical expertise.
